# Angiopoietin-Like Protein 4 Overexpression in Visceral Adipose Tissue from Obese Subjects with Impaired Glucose Metabolism and Relationship with Lipoprotein Lipase

**DOI:** 10.3390/ijms21197197

**Published:** 2020-09-29

**Authors:** Ilaria Barchetta, Caterina Chiappetta, Valentina Ceccarelli, Flavia Agata Cimini, Laura Bertoccini, Melania Gaggini, Claudio Di Cristofano, Gianfranco Silecchia, Andrea Lenzi, Frida Leonetti, Marco Giorgio Baroni, Amalia Gastaldelli, Maria Gisella Cavallo

**Affiliations:** 1 Department of Experimental Medicine, Sapienza University of Rome, 00161 Rome, Italy; ilaria.barchetta@uniroma1.it (I.B.); valentina.ceccarelli@uniroma1.it (V.C.); flaviaagata.cimini@uniroma1.it (F.A.C.); laura.bertoccini@uniroma1.it (L.B.); andrea.lenzi@uniroma1.it (A.L.); 2 Department of Medical-Surgical Sciences and Biotechnologies, Sapienza University, 04100 Latina, Italy; caterina.chiappetta@uniroma1.it (C.C.); claudio.dicristofano@uniroma1.it (C.D.C.); gianfranco.silecchia@uniroma1.it (G.S.); frida.leonetti@uniroma1.it (F.L.); 3 Cardiometabolic Risk Unit, Institute of Clinical Physiology, CNR, 56100 Pisa, Italy; mgaggini@ifc.cnr.it; 4 Department of Clinical Medicine, Public Health, Life and Environmental Sciences (MeSVA) University of L’Aquila, 67100 Coppito (AQ) Italy; marco.baroni@uniroma1.it; 5 IRCCS Neuromed, 86077 Pozzilli (Is), Italy

**Keywords:** ANGPTL4, lipoprotein lipase, lipids, adipose tissue, adipocyte, diabetes, glucose tolerance

## Abstract

Angiopoietin-like protein 4 (ANGPTL4) regulates lipid partitioning by inhibiting circulating and tissue lipoprotein lipase (LPL); ANGPTL4 loss-of-function variants improve insulin sensitivity and reduce type 2 diabetes (T2D) risk with mechanisms partially unknown. This study was designed to explore metabolic implications of differential ANGPTL4 and LPL expression in human adipose tissue (AT). We recruited eighty-eight obese individuals, with and without abnormal glucose metabolism (AGM), undergoing bariatric surgery; visceral AT (VAT) fragments were obtained intra-operatively and analyzed by immunohistochemistry and mRNA by rt-PCR. Data on hepatic ANGPTL4 mRNA were available for 40 participants. VAT ANGPTL4 expression was higher in AGM individuals than in those with normal glucose tolerance (NGT) and associated with VAT inflammation, insulin resistance, and presence of adipocyte size heterogeneity. Increased ANGPTL4 was associated with AGM with OR = 5.1 (95% C.I.: 1.2–23; *p* = 0.02) and AUROC = 0.76 (95% C.I.: 1.2–23; *p* < 0.001). High LPL was associated with the detection of homogeneous adipocyte size, reduced microvessel density, and higher HIF-1α levels and inversely correlated to blood transaminases. In conclusion, in obese individuals, VAT ANGPTL4 levels are increased in the presence of local inflammation and AGM. Conversely, higher LPL expression describes a condition of increased lipid storage in adipocytes, which may serve as a protective mechanism against ectopic fat accumulation and related metabolic disease in obesity.

## 1. Introduction

The angiopoietin-like proteins (ANGPTLs) are secreted factors structurally similar to angiopoietins; among them, ANGPTL3, 4, and 8 play a fundamental role in the regulation of lipid metabolism, mainly by binding to circulating lipoprotein lipase (LPL) and antagonizing its activity [1,2]. Thus, ANGPTLs prevent the LPL-mediated lipid hydrolysis—lipolysis—of triglyceride-rich lipoproteins (TRLs) in the bloodstream. Inversely, once LPL binds the endothelial membrane protein glycosylphosphatidylinositol-anchored high density lipoprotein-binding protein 1 (GPIHBP1), it becomes resistant to the inhibition exerted by ANGPTLs [3]. The overall effect of the selective LPL inhibition by ANGPTLs is the margination of TRLs in the capillary lumen, and the unloading of free fatty acids (FFAs) in vital organs, such as heart, skeletal muscle, and adipose tissue (AT), where FFAs serve as a fuel for energetic metabolism. The importance of ANGPTLs in the regulation of lipid metabolism has been established by genetic studies showing that genetic variations disrupting ANGPTL3 and ANGPTL4 function result in reduced triglyceride levels in humans [4,5,6]. 

Along with protecting from dyslipidemia, the inactivating variant E40K of the ANGPTL4 gene is associated with reduced risk of type 2 diabetes (T2D) [7]. Short-term treatment with monoclonal antibodies against ANGPTL3 and ANGPTL4 induces activation of LPL [7]; clinical trials have shown initial favorable effects with ANGPTL3 and ANGPTL4 antagonists on lipid profile and cardiovascular risk reduction [8]. Moreover, ANGPTL4 was shown to be involved in glucose metabolism, although findings in this regard are not conclusive, as ANGPTL4 overexpression in mice has been associated to either improved or impaired glucose tolerance in different studies [9,10,11]. Conversely, experimental ANGPTL4 knockout in mice improved insulin sensitivity and glucose homeostasis and, indeed, a role of ANGPTL4 antagonist in T2D therapy has been hypothesized [7].

The ANGPTL4-mediated regulation of metabolic processes is partially uncoupled from lipid partitioning [12] and involves glucose and insulin homeostasis; however, knowledge on the mechanisms mediating ANGPTL4 effects on human glucose regulation is still limited.

While ANGPTL3 is mainly produced by hepatocytes in the liver [13,14], ANGPTL4 is secreted and released by several tissues and organs, mostly by AT and liver [9]. Besides regulating circulating LPL, ANGPTL4 modulates intracellular lipolysis in tissues where is expressed; thus, local ANGPTL4 is critical for regulating tissue LPL activity [15,16,17,18,19]. AT represents one of the main sites of action of ANGPTL4 where its expression increases in the fasting state and in obesity, whereas it is suppressed by re-feeding and insulin in experimental models [16,17,18,19]. In the AT, ANGPTL4 promotes LPL intracellular cleavage in adipocytes, contributing to the regulation of local lipolysis in order to prevent excessive intra-adipocyte fat accumulation, cell hypertrophy, and hypoxia [15]. 

The evidence on ANGPTL4-mediated blood lipid regulation comes from animal studies, while data on the relationship between ANGPTL4 and LPL expression in AT and metabolic diseases in humans are very limited. Therefore, this study aimed to investigate ANGPTL4 and LPL expression in visceral AT (VAT) in relation to (i) local inflammation and dysfunction and (ii) clinical alterations, in human obesity. Moreover, since the vitamin D receptor (VDR) is involved in adipogenesis [20] and lipid accumulation in both liver [21] and AT [22] and a recent study demonstrated that VDR regulates ANGPTL4 gene expression in mice [23], we also explored VAT VDR expression in relation to local ANGPTL4 and LPL levels and the influence on the overall metabolic phenotype.

## 2. Results

Within our study participants, 50% fulfilled the criteria for metabolic syndrome (MS) diagnosis and 26% (23 out of 88 subjects) had abnormal glucose metabolism (AGM), as considered as impaired glucose regulation (*n*= 14/23, among whom *n* = 11 with impaired fasting glucose (IFG) and *n* = 3 with IFG + impaired glucose tolerance (IGT)) or T2D (*n*= 9/23). ANGPTL4 and LPL mRNA expression was detectable in the omental samples from all the study participants. Characteristics of the study population in relation to glucose tolerance are summarized in Table 1. 

### 2.1. VAT ANGPTL4 and LPL expression in Relation to Glucose Metabolism

**Figure 1:**
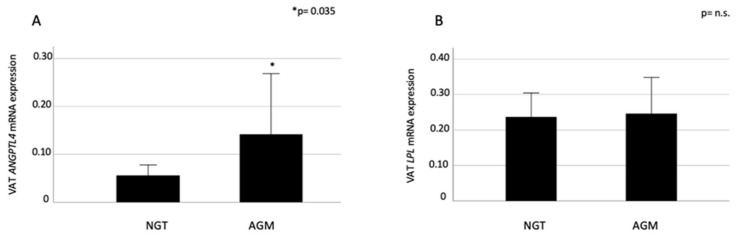
(**A**) Visceral adipose tissue angiopoietin-like protein 4 (VAT ANGPTL4) and lipoprotein lipase (LPL) (**B**) mRNA levels. Comparison between normal glucose tolerance (NGT) and abnormal glucose metabolism (AGM) individuals. * *P* value ≤ 0.05 is considered statistically significant. Data are shown as median (95% C.I.) values; arbitrary units. n.s., not significant.

ANGPTL4 expression levels were significantly higher in obese individuals with abnormal glucose metabolism than in those with normal glucose tolerance (*p* = 0.035, Mann–Whitney test, Figure 1). 

**Figure 2:**
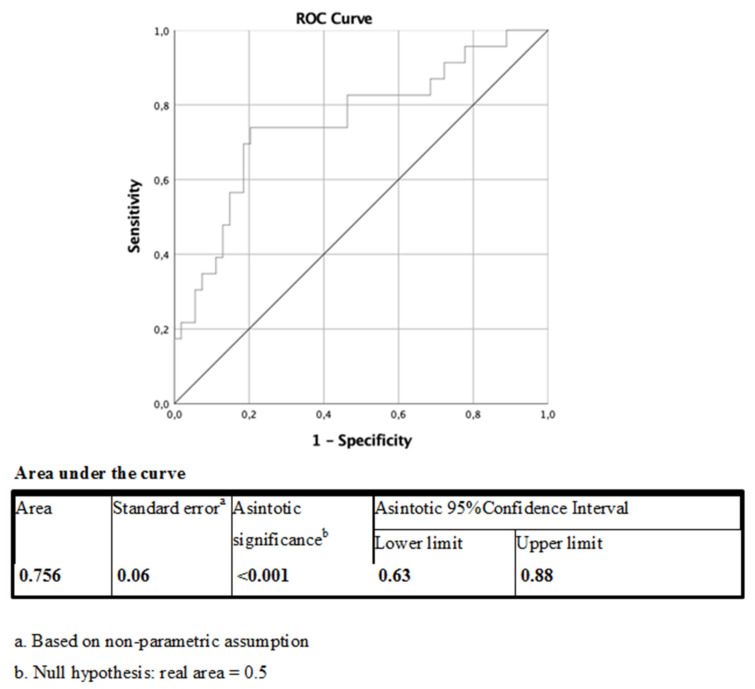
VAT ANGPTL4 receiver operating characteristic (ROC) curve for presence of abnormal glucose metabolism (AGM) corrected for age and sex.

VAT ANGPTL4 levels correlated negatively with high-density lipoprotein (HDL) cholesterol (r = −0.24; *p* = 0.048) and positively with fasting serum insulin (FSI) (*r* = 0.51; *p*= 0.008) and Homeostatic Model Assessment for Insulin Resistance (HOMA-IR) (*r* = 0.43; *p* = 0.029). On the contrary, no association was found between VAT ANGPTL4 and clinical indicators of obesity and ectopic fat accumulation, such as body mass index (BMI), waist circumference, and nonalcoholic fatty liver disease (NAFLD) (Table 2, column A). In our study population, higher VAT expression of ANGPTL4 was associated with the presence of AGM with an OR = 5.1 (95% C.I.: 1.2–23; *p* = 0.02; χ^2^ test applied) and with an AUC = 0.76 (95% C.I.: 0.63 – 0.88; *p* < 0.001) at the ROC curve adjusted for age and sex (Figure 2). 

VAT ANGPTL4 mRNA was not correlated with VAT LPL expression although increased LPL mRNA expression was associated with higher FSI (r = 0.46, *p* = 0.019) and insulin resistance (HOMA-IR: r = 0.44, *p*= 0.024). Negative correlation was found between VAT LPL levels and blood markers of liver injury, such as transaminases (aspartate aminotransferase (AST): r = − 0.23, *p* = 0.045; alanine aminotransferase (ALT): r = −0.36, *p*= 0.001), and gamma-glutamyl transpeptidase (GGT): r = −0.22, *p* = 0.045), regardless of NAFLD, which was diagnosed in 75% of participants undergoing liver biopsy (Table 2, column B). VAT LPL expression levels did not associate with AGM or MS in our study population (*p* = 0.92 and *p* = 0.63; χ^2^ tests applied).

### 2.2. Plasma ANGPTL4 Concentrations and Relation to ANGPTL4 and LPL Expression, and Glucose Metabolism

We measured plasma ANGPTL4 concentration to see if they were associated with either tissue expression. No relationship was found between either VAT ANGPTL4 or LPL mRNA levels and plasma ANGPTL4 concentrations. In the subgroup of subjects with liver biopsy (*n* = 40), we also investigated the association of circulating ANGPTL4 with hepatic ANGPTL4 expression, finding a weak positive correlation with increased hepatic ANGPTL4 expression (r = 0.32, *p* = 0.06).

Among metabolic parameters, plasma ANGPTL4 concentration correlated with higher total and LDL cholesterol levels (r = 0.30, *p* = 0.018; r = 0.27, *p* = 0.003, respectively).

### 2.3. VAT ANGPTL4 and LPL Expression in Relation to VAT Homeostasis

**Figure 3:**
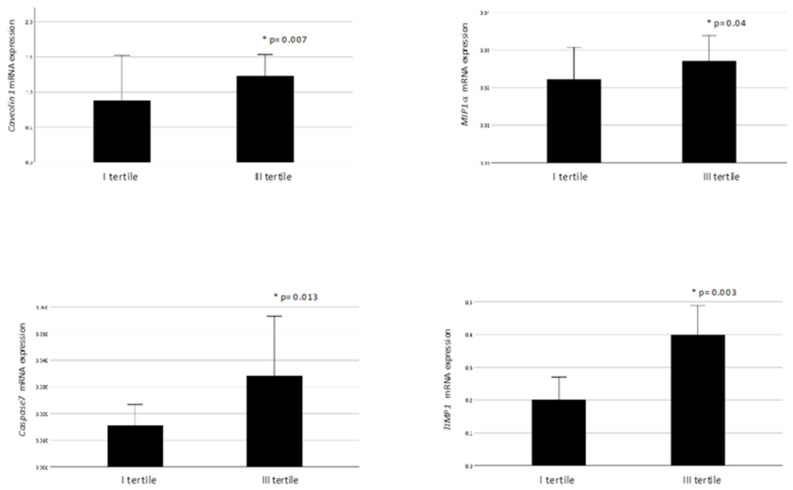
VAT Caveolin1, MIP1α, Caspase7, and TIMP1 mRNA expression in obese individuals with low (I tertile) or high (III tertile) VAT ANGPTL4 levels. Data are expressed as mean ± standard error. * *P* value ≤ 0.05 is considered statistically significant.

ANGPTL4 mRNA levels in the omental fat were significantly associated with the expression of local markers of inflammation, dysfunction, and insulin resistance. Noteworthy, obese individuals belonging to the highest tertile for VAT ANGPTL4 expression had higher Caveolin1 (*p* = 0.007), Caspase7 (*p* = 0.013), MIP−1α (*p* = 0.042), and TIMP−1 (*p* = 0.003) mRNA expression levels than those in the lowest ANGPTL4 subgroup (Figure 3). 

Linear correlates of VAT ANGPTL4 expression are reported in Table 2, column A. VAT LPL mRNA levels were also markedly increased in the presence of adipocyte hypoxia, as indicated by higher HIF−1α mRNA and lower microvascular density (CD34 positive cells per field at the immunohistochemistry), and impaired VAT energy balance, as expressed by greater PARP1 mRNA levels [24]. No association was found between LPL expression and markers of inflammation or apoptosis (Table 2, column B). 

### 2.4. VAT ANGPTL4 and LPL mRNA Levels in Relation to Adipocyte Heterogeneity

**Figure 4:**
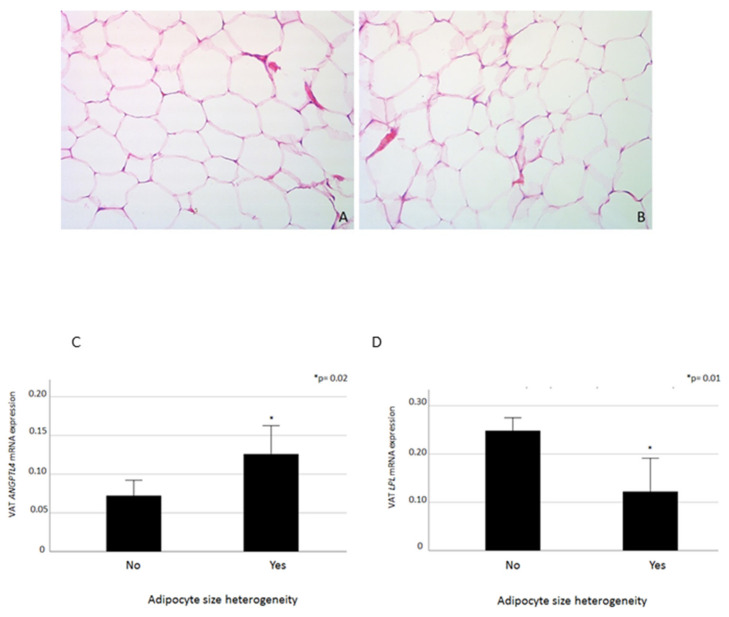
In the upper panel, two representative study participants with homogeneous (**A**) or heterogeneous (**B**) adipocyte size (400 × magnification). In the bottom panel, VAT ANGPTL4 (**C**) and LPL (**D**) mRNA expression levels in relation to the detection of adipocyte size heterogeneity at the immunohistochemistry. Data are expressed as mean ± standard error. * *p* Value ≤ 0.05 is considered statistically significant.

We observed marked heterogeneity of adipocyte size (see Figure 4 upper panel that shows two representative study participants with homogeneous (Figure 4A) or heterogeneous (Figure 4B) adipocyte size, as detected by difference in adipocyte dimensions at 400 × magnification in five fields at immunohistochemistry, in 10 out of 88 study participants. 

Obese individuals with heterogeneous adipocyte size had significantly higher fasting blood glucose (FBG) (118 ± 43.8 vs. 97.8 ± 17.4 mg/dl, *p* = 0.01), blood transaminases (AST: 41.4 ± 25.6 vs. 25 ± 10.9 IU/L, *p* = 0.05; ALT: 59.7 ± 36.1 vs. 32.9 ± 21.5 IU/L, *p* = 0.006; GGT: 44.3 ± 32.6 vs. 28.1 ± 24.4 IU/L, *p* = 0.035), while the VAT expression of ANGPTL4 was increased and LPL decreased, respectively (*p* ≤ 0.02, Figure 4 bottom panel). Moreover, size heterogeneity of VAT adipocytes was associated with increased Caspase7 mRNA expression (lower vs. upper median Caspase7 mRNA levels; *p* = 0.007, χ^2^ test applied). 

### 2.5. VAT ANGPTL4, LPL, and VDR mRNA Expression

**Figure 5:**
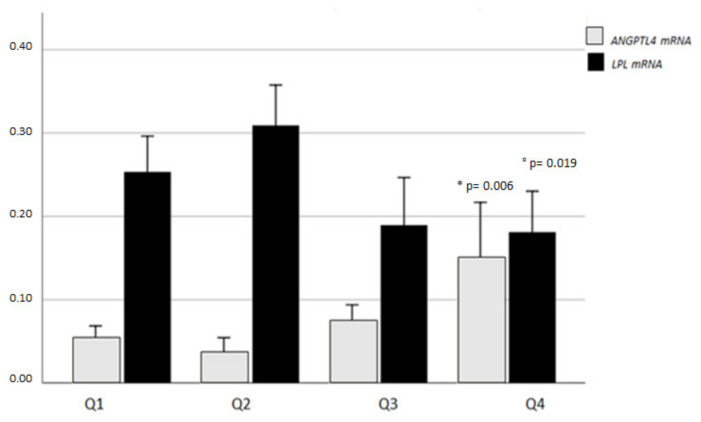
VAT ANGPTL4 and LPL mRNA expression levels across quartiles of vitamin D receptor (VDR) mRNA levels. Data are expressed as mean values ± standard error; *ANGPTL4 and °LPL inter-group multiple comparisons, *p* value ≤ 0.05 is considered statistically significant.

VDR is involved in the modulation of ANGPTL4. VAT ANGPTL4 levels were significantly increased and LPL decreased across quartiles of VDR expression (*p* = 0.006 and *p* = 0.019, respectively; Kruskal–Wallis inter-groups comparison test; Figure 5). 

VAT VDR mRNA levels correlated positively with ANGPTL4 (r = 0.47, *p* < 0.001) and negatively with LPL (r = −29, *p* = 0.009) levels at the bivariate analysis. Elevated VDR levels remained significantly associated with ANGPTL4 expression after adjustment for sex, age, BMI, waist circumference, HOMA-IR, and presence of AGM at the multivariable linear regression analysis (R^2^ = 0.72, *p* = 0.037; Table 3).

## 3. Discussion

This study was designed to explore VAT ANGPTL4 and LPL expression levels in human obesity in relation to adipose tissue inflammation, impairment in glucose metabolism, and clinical outcomes. We showed that VAT ANGPTL4 is increased in obese subjects with impaired glucose regulation and T2D and with insulin resistance. Omental fat from obese individuals with higher ANGPTL4 displayed signs of inflammation and meta-inflammation, i.e., apoptosis, cell stress, and matrix rearrangement markers, known to be involved in the alteration of insulin sensitivity and development of metabolic diseases [25,26,27]. Conversely, LPL expression in omental fat varied in relation to tissue plasticity; we observed higher LPL levels in the presence of homogeneous adipocyte size and no signs of inflammation, whereas LPL levels were low in inflamed, inhomogeneous adipose tissue. LPL is considered the “gatekeeper of the adipocyte” [28] and is required for the hydrolysis of circulating triglycerides and efficient FFA uptake into the adipocytes [29]. Indeed, AT LPL overexpression has been proposed as an adaptive pattern to over-nutrition and might protect, to some extent, from consequences of excessive VAT lipolysis, lipids efflux, and deposition in non-adipose organs [30,31]. 

To date, data on ANGPTL4 and LPL expression in human adipose tissue are limited to few observations [16,32]. In particular, Dijk and collaborators reported positive correlation between ANGPTL4 and LPL mRNA levels in omental and subcutaneous AT, but not in mesenteric fat [16]. Very recently, McCulloch and coinvestigators [32] found increased ANGPTL4 expression in subcutaneous AT from obese patients with T2D in comparison to those without diabetes; after weight loss surgery, AT ANGPTL4 levels declined only in diabetic individuals, but no data on tissue LPL expression were available. In our cohort, VAT LPL and ANGPTL4 levels were not correlated, indicating a more intricate regulation of this enzyme in the adipose tissue [33].

In our study, we demonstrated the existence of a strong association between high LPL expression and signs of visceral adipose tissue hypoxia, as expressed by greater HIF-1α mRNA levels and reduced number of CD34 positive cells, representative of altered microvascular density. LPL overexpression was also associated with higher expression levels of the poly(ADP-ribose) polymerase (PARP) 1, which regulates adipose energy balance and adipogenesis in experimental models [24]. Although VAT LPL overexpression was associated with signs of hypoxic state, this was not accompanied by signs of cell apoptosis or inflammation. 

Previous investigations have demonstrated that tissue hypoxia represents one of the first pathophysiological changes in condition of caloric overload [34,35,36]. In rodents, HIF−1α upregulation starts early after the administration of a high-fat diet, before inflammation and insulin resistance develop [35,36], and HIF−1α induces ANGPTL4 expression, likely as a compensatory mechanism to limit adipocyte expansion [37,38,39]. However, limited lipid storage in the AT may favor ectopic fat accumulation in non-adipose tissues, which, in turn, negatively affects glucose homeostasis. 

In our study population, LPL overexpression did not depict unfavorable metabolic outcomes, such as T2D, and was instead negatively correlated with markers of hepatic damage. 

The existence of an uncoupling between fat expansion and impaired glucose metabolism has been demonstrated in ANGPTL4 knockout mice, where, despite AT enlargement, loss of ANGPTL4 improved glucose tolerance with mechanisms independent from β cell insulin secretion [12]. 

In our study, as in previous reports [12,32], ANGPTL4 overexpression is associated with the presence of signs of AT inflammation; however, in mice fed a high-fat diet, the lack of ANGPTL4 positively impacts on systemic glucose regulation without improving VAT inflammatory state [40]. 

Thus, the effect of ANGPTL4 on glucose metabolism seems to be directly ascribed to its inhibitory effect on LPL, which translates into impaired lipid trafficking and accumulation, rather than being mediated by improved insulin secretion and/or VAT inflammation.

Our study demonstrates that VAT ANGPTL4 overexpression is associated with impaired local lipid metabolism also in humans. Among our study participants, a subgroup of patients presented marked adipocyte size heterogeneity, which might result from large adipocyte apoptosis, replacement with small-size adipocytes, tissue hyperplasia, and remodeling, as described in animal models [36]. Individuals with VAT heterogeneity in adipocyte size had significantly increased ANGPTL4 and reduced LPL levels, along with worse metabolic profile, as expressed by higher FBG and transaminases, in comparison to those with homogenous adipocyte size. 

ANGPTL4 has been recently shown to be a transcriptional target of the VDR in intestinal cells, and to mediate the VDR effects on lipid metabolism and fat accumulation in mice [21]. Indeed, we demonstrated the existence of an independent relationship between VDR and ANGPTL4 expression in human VAT, regardless of potential confounders such as age, sex, body adiposity, and metabolic disease.

We could not link, in our population of obese subjects, circulating ANGPTL4 concentration with either VAT ANGPTL4 expression or presence of impaired glucose metabolism. Of note, we found weak correlation between plasma and liver ANGPTL4, in line with previous studies reporting significant contribution of hepatic secretion to overall circulating ANGPTL4 levels [9]. The identification of an association between impaired glucose metabolism and ANGPTL4 expression in VAT—rather than its circulating levels—points towards to a central role of AT ANGPTL4/LPL pathway in controlling glucose homeostasis in humans. 

This study has several limits as follows. (1) The expression of ANGPTL4 and LPL was measured only in VAT and not subcutaneous fat. However, previous studies have shown that accumulation of fat in VAT is a risk factor for metabolic diseases, whereas expansion of subcutaneous adipose tissue is associated with a better metabolic profile [41,42]. (2) We did not measure FFA concentrations; however, FFA levels mainly reflect FFA released by the subcutaneous adipose tissue, whereas FFAs released by the VAT, which is hyperlipolytic compared to subcutaneous adipose tissue [41], are taken up by the liver.

In conclusion, this study demonstrates that VAT ANGPTL4 overexpression is associated with local metabolic impairment and inflammation and predicts impaired glucose regulation and type 2 diabetes in obese individuals. Conversely, higher LPL describes a condition of increased lipid storage in adipocytes, which may serve as a protective mechanism against ectopic fat distribution and related metabolic disease in obesity. Our findings provide insights into the understanding of ANGPTL4 and LPL pathways in human adipose tissue and add knowledge on ANGPTL4 involvement in glucose regulation in obesity.

## 4. Materials and Methods 

### 4.1. Study Population

For the purposes of this cross-sectional investigation, eighty-eight consecutive male and female obese subjects were recruited among those undergoing pre-intervention metabolic evaluations for bariatric surgery at Sapienza University of Rome, Italy. Patients with body mass index (BMI) ≥ 40 kg/m^2^ or BMI: 35–40 kg/m^2^ in presence of comorbidities were considered eligible for this study; the inclusion and exclusion criteria followed those reported in the International guidelines for sleeve gastrectomy surgery [43]. For each eligible subject we collected medical history, and measured routine clinical and experimental parameters on fasting blood samples. Weight and height were measured with light clothes and without shoes and waist circumference (cm) was measured midway between the 12th rib and the iliac crest. Systemic systolic and diastolic blood pressure (SBP, DBP, mmHg) were measured after 5-minute resting three times and reported as the average of the second and third measurements. 

Abnormal glucose metabolism (AGM) was diagnosed in presence of impaired fasting glucose (IFG) and/or impaired glucose tolerance (IGT) or T2D, defined according to the American Diabetes Association 2020 criteria [44]; metabolic syndrome (MS) was diagnosed based on the NCEP-ATP III definition [45]. 

### 4.2. Laboratory Tests

Study participants underwent fasting blood sampling for routine biochemistry measurement, including: blood glucose (FBG, mg/dL), glycosylated haemoglobin (HbA1c, mmol/mol, %), aspartate aminotransferase (AST, IU/L), alanine aminotransferase (ALT, IU/L) gamma-glutamyl transpeptidase (GGT, mg/dL), uric acids (mg/dL), creatinine (mg/dL), total cholesterol (mg/dL), high-density lipoprotein cholesterol (HDL, mg/dL) and triglycerides (mg/dL), all assessed by centralized standard methods. Low-density lipoprotein (LDL) cholesterol value was calculated with the Friedewald formula. Fasting serum insulin (FSI, IU/mL) was measured by radioimmunoassay (ADVIA Insulin Ready Pack 100; Bayer Diagnostics, Milan, Italy; intra- and inter-assay coefficients of variation <5%). Plasma ANGPTL4 concentrations were measured using the Luminex technology (Merck KGaA on MAGPIX^®^ -MILLIPLEX^®^, Darmstadt, Germany). 

### 4.3. Vat and Liver Biopsy Collection, Immunohistochemistry and Gene Expression Analyses

For the purposes of this study, VAT samples were collected intra-operatively from omental fat tissue. For immunohistochemistry studies, consecutive sections (3um) were deparaffinized and rehydrated in graded ethanol, then sections were stained with haematoxylin and eosin and Masson’s trichrome for histological evaluations and assessing the fibrosis respectively. Fast green FCF/Sirius stainings were carried out for collagen fibers detection and CD68 and CD34 monoclonal antibodies were used to quantify macrophages and micro-vessel density, respectively. Results were expressed as previously described [46]. Real time quantitative PCR for CAV1 (gene ID: 857), MIP1A (gene ID: 6348), TIMP1 (gene ID: 7076), CASP7 (gene ID: 836), HIF-1α (gene ID: 3091), PARP1 (gene ID: 142), LPL (gene ID: 4023), ANGPTL4 (gene ID: 51129), VDR (gene ID: 7421) mRNA was performed as described in our previous study [46]. 

ANGPTL4 mRNA expression was measured in liver biopsy taken during surgery (available for 40 out of 88 participants). Experimental procedures for liver gene expression analyses are detailed elsewhere [14]. Liver histology was reported based on Brunt score [47], the presence of NASH was diagnosed by the joint presence of steatosis, ballooning and lobular inflammation; NAFLD activity score (NAS) was calculated as the sum of scores for steatosis, lobular inflammation, and ballooning [48]; fibrosis was quantified on the basis of the NASH Clinical Research Network Scoring System Definition [48]. Two pathologists, blinded to the experimental protocol and participants’ identity, performed all the analyses. 

### 4.4. Statistics

Statistical analyses have been performed using SPSS package version 25. Values are reported as mean ± SD, median (95% confidence interval, C.I.) or percentage, as appropriate. Mann-Whitney test for continuous variables and χ^2^ test for categorical parameters were used for comparisons between two independent groups; comparisons between > 2 subgroups were performed by Kruskal Wallis test. Bivariate correlations were explored by Spearman’s r coefficients. The independence of the association between VAT ANGPTL4 expression and AGM, was explored by a sex-, age-forced multivariable logistic regression model including VAT ANGPTL4 mRNA levels and potential confounders. The predictive value of ANGPTL4 expression levels on the presence of AGM was further evaluated by receiver operating characteristic (ROC) curve adjusted for sex and age. Multivariable linear regression model was built to test the association between VAT VDR and ANGPTL4 levels entering sex, age and metabolic parameters as covariates. A two-tailed p value < 0.05 was considered statistically significant, with a 95% confidence interval. 

### 4.5. Ethics Standards

The study protocol was reviewed and approved by the local Ethics Committee (approval number 3550, 26 February 2015); the study was conducted in conformance with the Helsinki Declaration. Informed written consent was obtained from all participants before the study procedures.

## Figures and Tables

**Table 1:** Characteristics of the entire study population and in relation to glucose tolerance.

	All Participants; *n* = 88	NGT; *n*= 65	AGM; *n* = 23	*p*-value
Age (years)	44.1 ± 9.9	42.4 ± 9.8	48.4 ± 8.9	0.017
Sex (male/female)	26/62	17/48	9/14	0.13^1^
BMI (kg/m^2^)	42.4 ± 4.8	42 ± 4.9	43.5 ± 4.7	0.24
Waist circumference (cm)	126.2 ± 13	125.4 ± 14.3	128.9 ± 9.3	0.36
SBP (mmHg)	129.4 ± 14	129.2 ± 13.4	130.7 ± 15.7	0.67
DBP (mmHg)	84.2 ± 14	84.3 ± 7.7	84 ± 23	0.92
Total cholesterol (mg/dl)	194.5 ± 32.2	196.3 ± 31	188.3 ± 32	0.34
HDL-C (mg/dl)	47.5 ± 10.9	49.3 ± 11	42.9 ± 9.8	0.02
LDL-C (mg/dl)	118.5 ± 31.2	120.6 ± 31	111.7 ± 30	0.28
Triglycerides (mg/dl)	138.8 ± 62	130.5 ± 58.7	158 ± 70.3	0.039
FBG (mg/dl)	100 ± 23	89.9 ± 5.3	126.8 ± 29.2	<0.001
HbA1c - mmol/mol; %	38.3 ± 8.8; 5.6 ± 0.7	35.2 ± 3; 5.3 ± 0.3	49 ± 13.8; 6.1 ± 1	0.003
AST (IU/l)	27.6 ± 14.4	25.5 ± 11.5	33 ± 18.9	0.06
ALT (IU/l)	36.2 ± 25	31.8 ± 23	46.6 ± 28	0.005
GGT (IU/l)	30 ± 25.6	25.5 ± 13	42.8 ± 42.6	0.06
Uric acid (mg/dl)	5.6 ± 1.4	5.4 ± 1	6.0 ± 1.95	0.56
Serum creatinine (mg/dl)	0.76 ± 0.1	0.74 ± 0.1	0.79 ± 0.2	0.28
Plasma ANGPTL4 (pg/mL)	174 ± 64.8	175.6 ± 67	160.2 ± 56.8	0.33

Values are expressed as mean ± SD; *p*-values are relative to comparison between the NGT and AGM subgroups, Student’s T test applied; ^1^ χ^2^ test applied. AGM, abnormal glucose metabolism; NGT, normal glucose tolerance; BMI, body mass index; SBP, sistolic blood pressure; DBP, diastolic blood pressure; HDL-C, high-density lipoprotein cholesterol; LDL-C, low-density lipoprotein cholesterol; FBG, fasting blood glucose; HbA1c, glycosylated haemoglobin; AST, aspartate aminotransferase; ALT, alanine aminotransferase; GGT, gamma-glutamyl transpeptidase; ANGPTL4, angiopoietin-like protein 4.

**Table 2:** Bivariate correlation analyses between (A) ANGPTL4 and (B) LPL mRNA expression and covariates.

	ANGPTL4 mRNA		LPL mRNA	
	r Coefficient	*p*-Value	r Coefficient	*p*-Value
Age	−0.14	0.22	−0.15	0.18
BMI	−0.16	0.18	0.07	0.56
Waist circumference	0.10	0.44	−0.12	0.36
FBG	0.10	0.39	−0.04	0.97
FSI	0.51	0.008	0.46	0.019
HbA1c	0.13	0.29	0.21	0.20
Total Cholesterol	0.06	0.63	−0.15	0.22
HDL	−0.24	0.048	0.04	0.73
LDL	0.12	0.34	−0.18	0.13
Triglycerides	0.08	0.48	−0.06	0.61
AST	−0.07	0.56	−0.23	0.045
ALT	0.06	0.59	−0.36	0.001
GGT	−0.11	0.12	−0.22	0.045
HOMA-IR	0.43	0.029	0.44	0.024
LPL	0.13	0.22	-	-
Caveolin1	0.25	0.018	0.18	0.10
MIP-1α	0.22	0.043	0.07	0.49
HIF-1α	0.10	0.35	0.38	<0.001
TIMP1	0.30	0.005	0.16	0.14
Caspase7	0.29	0.006	−0.16	0.13
PARP1	0.06	0.57	0.23	0.03
Plasma ANGPTL4	0.03	0.81	−0.001	0.99

ANGPTL4 and LPL levels are considered as continuous variables; r = Spearman’s coefficient. FBG, fasting blood glucose; FSI, fasting blood insulin; HbA1c, glycosylated haemoglobin; HDL-C, high-density lipoprotein cholesterol; LDL-C, low-density lipoprotein cholesterol; AST, aspartate aminotransferase; ALT, alanine aminotransferase; GGT, gamma-glutamyl transpeptidase; HOMA-IR, homeostatic model assessment for insulin resistance; LPL, lipoprotein lipase; ANGPTL4, angiopoietin-like protein 4.

**Table 3:** Multivariable linear regression analysis. VAT ANGPTL4 expression is the dependent variable.

	Unstandardized Coefficients		Standardized Coefficient	t	*p*-Value
	B	Standard Error	Beta		
(Constant)	5.69	5.07		1.12	0.31
VAT VDR mRNA	2.58	0.91	1.28	2.82	0.037
Age	−0.043	0.03	−0.44	−1.52	0.19
Sex	−0.010	0.63	−0.005	−0.016	0.98
BMI	−0.075	0.057	0.47	−1.33	0.19
Waist circumference	0.28	0.31	0.287	0.92	0.40
HOMA-IR	0.53	0.72	0.85	1.94	0.11
Presence of AGM	−0.24	1.38	−1.99	−1.77	0.137

VAT, visceral adipose tissue; VDR, vitamin D receptor; BMI, body mass index; HOMA-IR, homeostatic model assessment for insulin resistance; abnormal glucose metabolism.

## Abbreviations

AGM	abnormal glucose metabolism
ALT	alanine aminotransferase
ANGPTL	angiopoietin-like protein
AST	aspartate aminotransferase
AT	adipose tissue
BMI	body mass index
DBP	diastolic blood pressure
FBG	fasting blood glucose
FBI	fasting blood insulin
FFA	free fatty acid
GGT	gamma-glutamyl transpeptidase
HbA1c	glycosylated hemoglobin
HDL	high-density lipoprotein
IFG	impaired fasting glucose
IGT	impaired glucose tolerance
LPL	lipoprotein lipase
MS	metabolic syndrome
NAFLD	nonalcoholic fatty liver disease
NAS	NAFLD Activity Score
NASH	nonalcoholic steatohepatitis
NGT	normal glucose tolerance
SBP	systolic blood pressure
T2D	type 2 diabetes
VDR	vitamin D receptor
